# A future perspective for regenerative medicine: understanding the concept of vibrational medicine

**DOI:** 10.4155/fsoa-2017-0097

**Published:** 2018-01-05

**Authors:** Kavita Beri

**Affiliations:** 1 Department of Biomedical Engineering, Rutgers, The State University of New Jersey, 599 Taylor Road Piscataway, NJ 08854, USA; 2 Medical Director and Founder Beri Esthetique: Mind Body Skin; 3 Center for Dermal Research, New Jersey Center for Biomaterials, Rutgers – The State University of New Jersey, 145 Bevier Road Piscataway, NJ 08854, USA

**Keywords:** anti-aging, heal, life force, regeneration, stem cells, vibrational medicine

Regenerative Medicine has reached a new frontier, and understanding the concept of energy medicine and vibration science in relation to body physiology could provide us with a deeper understanding of the cellular processes that govern healing and perhaps unlock new avenues in tissue engineering. This paper is written with the intention of introducing vibration medicine as a new dimension to the expanding field of regenerative medicine.

In the expanding field of regenerative medicine, new avenues of research in the field of energy medicine and vibration science could unlock a deeper understanding of body healing mechanisms and the regeneration of tissue that could be applied for new therapies. This paper is written to influence research in the field of vibrational science in conjunction with tissue engineering.

In the rapidly expanding field of regenerative medicine, the focus is on replicating the inherent natural mechanisms in the body to perfection. The focus is on developing techniques that can mimic the perfectly engineered autoregulation's healing, cell division, cell maturation and homeostasis in tissue. We have come a long way in new avenues to replace damaged tissue, deliver stem cells in areas of degeneration, help heal skin wounds and reduce aging [1]. There is no doubt that the basis of creating perfection in the regenerative techniques is by a thorough understanding of the basic physiology of the human body and perhaps considerations beyond our current understanding of basic physiology. All theory brings us to question where the single cell programmed itself to become the entire organism, or the ‘origin’. How close can we get to that origin, or the ‘force’ as most ancient texts refer to the energy that is the life force behind all processes in the body?

This leaves scientists with the interesting aspect of going back to understanding how this fascinating ‘life force’ can be harnessed and perhaps applied to the expanding field of regenerative medicine. This article seeks to inspire innovation in the field by summarizing what research has been performed in vibrational and energy healing in terms of regenerative medicine, and perhaps evolve them into newer applications of tissue engineering and regenerative therapies to reach the perfection we desire.

## The concept of Biofield energy: mechanical & subtle energies in the evolution of vibrational medicine

The emerging science of the ‘Biofield’ aims to provide a scientific foundation for homodynamic regulation of living systems. By probing deeper into this field we arrive at an improved understanding of the foundations of biology and body physiology as well as the concept of Energy Medicine [2].

As per author and physician scientist Dr Richard Gerber [3], the Newtonian model of medical thinking sees the human physiological and psychological behavior as dependent upon structural hardware of the brain and body. However, the Newtonian mechanistic viewpoint of life is only an approximation of reality. In a machine, the function of the whole can be predicted by the sum of its parts; however, in reality, the human body is not a sum of its individual components; there is the force that ‘animates.’ Once that force leaves the body, as in after death, the body organs slowly deteriorate. A modern and more recent perspective of the life force has involved looking at it as a vibration science based on Einstein's paradigm. This concept sees human beings as a network of complex energy fields that interface with physical and cellular systems. Vibrational medicine uses specialized forms of energy to positively affect those energetic systems that may be out of balance due to diseased states [3].

Subtle energies are the foundation of the consideration of ethereal bodies [3], and not just the anatomical/physiological maps of what we consider in modern medicine. Ethereal bodies include maps and meridians of energy flow and chakra points. The lines of energy that can be mapped are called Nadis in Ayurveda and Qi energy lines in acupuncture. Over the decades, many forms of healing have looked at harnessing these subtle energies, such as Homeopathy, Reiki, reflexology, Yoga Therapy, acupuncture and Ayurveda. These are practiced today, and have a strong following of people that absolutely believe in these methods of healing [3].

## From ancient medicine to the evolution of vibrational medicine

### Various popular ancient medicine practices have utilized ethereal body maps to understand energy flow within the body

In the Chinese belief, the life force is the Qi energy (breath), also known as the ‘Yang’, which mixes with the blood, or the ‘Yin energy’, and creates the balance for life [4,5]. Per this belief the imbalance of the Yin and the Yang creates the disease process. Qi travels throughout the body along ‘Meridians’, or special pathways. Acupuncture is based on the belief of inserting needles along certain energy points that would help improve and balance the Qi energy in proper flow [6,7]. In this vein, one study reported that endomorphin-1, β endorphin, encephalin and serotonin levels increased in plasma and brain tissue through acupuncture application, which resulted in analgesia, sedation and recovery in motor functions [8].

Originating in India, Ayurveda is one of the ancient yet living health traditions [9]. Life in Ayurveda is conceived as the union of the body, senses, mind and spirit. Ayurveda is based on looking at the body as five elements that constitute the material component structure through which the prana (breath) energy flows through the ‘Nadis’ and animates the body. The five elements are water, earth, fire, ether and air [10]. The body is assessed on the basis of its Doshas; that is, expressed as *Prakriti* (phenotype), which broadly means a body type or individual nature. One clinical study showed a strong correlation between HLA alleles and Ayurvedic *Prakriti* type [11]. Later, it was also hypothesized that different *Prakritis* may possess different drug metabolism rates associated ayurvedic ‘prakriti’ types, suggesting a strong correlation to physiology and pathophysiology [12].

The practice of Yoga is said to use breath (Prana) energy to purify the body along with certain postures that have physiologic benefits to the body [13]. Several clinical trials based on assessing changes in the inflammatory markers in the body in response to the yogic lifestyle have shown improvement in overall health, and recovery from cancer and mental health issues [13–16]. This research demonstrates a clear link between these practices and health, and thus suggests their use might be widened to improve other areas of medicine, such as regenerative anti-aging.

## Evidence-based literature looking at subtle energies, mind–body connection & electromagnetic fields

A New York-based orthopedic surgeon in his research reported the presence of electromagnetic waves around a healing tissue. Becker's [17] original work observed the phenomena known as the ‘current of injury’. This is the electrical potential that can be measured from an amputated stump in an animal. When surgically removing a limb from an animal in the laboratory, the changes of the electrical patterns at the stump could be measured over several days during the healing and repair process [17]. More recently a paper by Hunckler *et al*. discussed how electrostimulation of wounds has been shown to be a promising treatment for accelerating regeneration in wounds [18]. Similarly, an insight into how the clock mechanisms of the stem cells in the skin respond to environmental cues in the shift in light energy are interesting insights into the energetic influences of the cell [19]. In the field of cosmetics, and antiaging of skin with the use of new-age laser devices, a photomechanical and photo thermal effect of the extracellular matrix of the dermis has been shown to cause neocollagenesis, thermal/photo-mechanical ‘energy’ that is an another example of the concept of vibration [20]. New ultrasound-based drug delivery systems use sound energy to deliver topical preparations deeper by influencing cellular ionic balance [21].

A recent study was designed to explore the impact of Yoga and Meditation based lifestyle intervention (YMLI) on cellular aging in apparently healthy individuals [22]. During this 12-week prospective, open-label, single arm exploratory study, 96 apparently healthy individuals were enrolled to receive YMLI. The primary end points were assessment of the change in levels of cardinal biomarkers of cellular aging in blood from baseline to week 12, which included DNA damage marker 8-hydroxy-2′-deoxyguanosine (8-OH2dG), oxidative stress markers reactive oxygen species and total antioxidant capacity, and telomere attrition markers telomere length and telomerase activity. The secondary end points were assessment of metabotrophic blood biomarkers associated with cellular aging, which included cortisol, β-endorphin, IL-6, BDNF and sirtuin-1. After 12 weeks of YMLI, there were significant improvements in both the cardinal biomarkers of cellular aging and the metabotrophic biomarkers influencing cellular aging compared with baseline values. The mean levels of 8-OH2dG, reactive oxygen species, cortisol and IL-6 were significantly lower and mean levels of total antioxidant capacity, telomerase activity, β-endorphin, BDNF and sirtuin-1 were significantly increased (all values) post YMLI. The mean level of telomere length was increased but the finding was not significant. YMLI significantly reduced the rate of cellular aging in apparently healthy population [22].

The healing power of thought is a concept that is looked at with clinical research done on meditation. A recent observation by scientist Hankey A has documented improvement in sensory acuity, perceptual style but also cognitive function, indicating stabilization of aspects of attentional awareness when comparing advanced practitioners of Tibetan Buddhist meditation in remote regions of the Himalayas, with established results on long-term practitioners of the Transcendental Meditation programs [23]. In another recent publication, the authors puts forth the hypothesis that mental phenomena are connected with thermodynamic properties of a large neural network [24].

In various cell migration studies, traction is a measurable unit, and traction measurements have been shown to be quantified. The ‘force’ of both traction and contraction can be measured [25]. The impacts of sound, heat and light on cells have been studied and documented, and cellular changes have been reported. If external forces have an impact on living cells, and we believe in the theory of equal and opposite reactions, there will be an internal force that is acting to negate the external force, and the balance of the two forces perhaps leads to cellular changes. Studies show specific electromagnetic field frequencies enhance human bone marrow stromal cell (hSSC/BMSC) adherence, proliferation, differentiation and viability, all of which play a key role in the use of hSSCs/BMSCs for tissue engineering in recreating cartilage tissue, bone and muscle [26]. For decades it has been reported that energy fields can play important roles in determining cell differentiation, migration, adhesion and even wound healing. Combinations of electric field, magnetic field and electromagnetic field techniques have revealed new and exciting explanations for how cells move and adhere to surfaces; how the migration of multiple cells are coordinated and regulated; and how cells interact with neighboring cells and to changes in their microenvironment [27].

## Conclusion & future perspective: vibrational medicine & its future with regenerative medicine

As a scientist, one's journey involves unlocking secrets within the realm of the known body and cellular processes. We have only begun to understand pathways and cellular interfaces that have governed life for thousands of years. What lies beyond the concepts of known facts are the possible discoveries of unseen forces that regulate the many regulatory processes in the body. Learning from these ancient concepts of subtle energies brings to light possible explanations for cell behavior, autoregulation, tissue healing and understanding of forces beyond the reach of quantitate measurements as factors that play a role in regeneration and maintenance. As modern-day scientists, with modern equipment, can we take ancient concepts and utilize them?

The goal of this opinion article is to inspire more regenerative medicine research into the field of vibrational medicine, perhaps by looking to quantify the pressure used in acupuncture needles to create a proper flow of Qi for a patient to see a result [28], or measuring inflammatory markers after channeling Chakra healing and Chakra energy. Perhaps also to understand and teach the ethereal maps of the body as a mainstream course in medicine and basic science so that we can have a foundation knowledge of all possible ways of looking at the human body as a ‘multidimensional being’ [29]. We can then arrive at more modern applications of ancient knowledge that is current with our knowledge of the stem cells, wound healing and tissue engineering. There should be a meeting point where alternative healing and mainstream science merge and learn from each other. As Faculty, I applaud conferences that provide a platform for such merger, like the International Society of Regenerative Medicine [30]. The expansion in the regenerative medicine field will reach a point where controlling the internal forces and interactions between cells would be essential to perfect the healing technologies and perhaps slowing down the process of aging. But for that we must have a strong knowledge and foundation base of ancient techniques as scientists. The next wave of regenerative medicine scientist might be ‘the spiritual scientist’, focused on understanding energy flow in human physiology.

**Executive summary**

Introducing the concept of vibration science and energy mechanics in the body

- Ancient practices that focus on body energy and vibrational healing.
- Evidence based understanding and historical over view of vibration and the biofield to date.
- Conclusion and future perspective: incorporating vibration medicine into regenerative medicine and tissue engineering.
